# The chloroplasts genomic analyses of four specific *Caragana* species

**DOI:** 10.1371/journal.pone.0272990

**Published:** 2022-09-01

**Authors:** Maohua Yuan, Xianmei Yin, Bixing Gao, Rui Gu, Guihua Jiang

**Affiliations:** 1 College of Pharmacy, Chendu University of Traditional Chinese Medicine, Chendu, China; 2 Traditional Chinese Medicine Detection, Leshan Food and Drug Inspection and Testing Center, Leshan, China; 3 Sichuan Institute for Drug Control, Key Laboratory of Quality Evaluation of Chinese Patent Medicines, Chengdu, China; 4 Key Laboratory of Standardization of Chinese Medicines of Ministry of Education, State Key Laboratory of Characteristic Chinese Medicine Resources in Southwest China, School of Pharmacy, Chengdu University of Traditional Chinese Medicine, Chengdu, China; 5 School of Ethnic Medicine, Chengdu University of TCM, Chengdu, China; Sant Baba Bhag Singh University, INDIA

## Abstract

**Background:**

Many species of the genus *Caragana* have been used as wind prevention and sand fixation plants. They are also important traditional Chinese medicine, and ethnic medicine resource plant. Thus, chloroplast genomes (cp-genome) of some of these important species must be studied.

**Methods:**

In this study, we analyzed the chloroplast genomes of *C*. *jubata*, *C*. *erinacea*, *C*. *opulens*, and *C*. *bicolor*, including their structure, repeat sequences, mutation sites, and phylogeny.

**Results:**

The size of the chloroplast genomes was between 127,862 and 132,780 bp, and such genomes contained 112 genes (30 tRNA, 4 rRNA, and 78 protein-coding genes), 43 of which were photosynthesis-related genes. The total guanine + cytosine (G+C) content of four *Caragana* species was between 34.49% and 35.15%. The four *Caragana* species all lacked inverted repeats and can be classified as inverted repeat-lacking clade (IRLC). Of the anticipated genes of the four chloroplast genomes, introns were discovered in 17 genes, most of which were inserted by one intron. A total of 50 interspersed repeated sequences (IRSs) were found among them, 58, 29, 61, and 74 simple sequences repeats were found in *C*. *jubata*, *C*. *bicolor*, *C*. *opulens*, and *C*. *erinacea*, respectively. Analyses of sequence divergence showed that some intergenic regions (between *trnK-UUU* and *rbcl*; *trnF*-*GAA* and *ndhJ*; *trnL*-*CAA* and *trnT*-*UGU*; *rpoB* and *trnC*-*GCA*; *petA* and *psbL*; *psbE* and *pebL*; and sequences of *rpoC*, *ycf1*, and *ycf2*) exhibited a high degree of variations. A phylogenetic tree of eight *Caragana* species and another 10 legume species was reconstructed using full sequences of the chloroplast genome.

**Conclusions:**

(1) Chloroplast genomes can be used for the identification and classification of *Caragana* species. (2) The four *Caragana* species have highly similar cpDNA G+C content. (3) IRS analysis of the chloroplast genomes showed that these four species, similar to the chloroplast genome of most legumes, lost IRLC regions. (4) Comparative cp-genomic analysis suggested that the cp genome structure of the *Caragana* genus was well conserved in highly variable regions, which can be used to exploit markers for the identification of *Caragana* species and further phylogenetic study. (5) Results of phylogenetic analyses were in accordance with the current taxonomic status of *Caragana*. The phylogenetic relationship of *Caragana* species was partially consistent with elevation and geographical distribution.

## 1. Introduction

About 100 *Caragana* species exist worldwide. Among the species in the arid and semi-arid regions of Asia and Europe, 66 species (32 endemic) can be found in China [1]. The genus *Caragana* is a deciduous shrub with a wide range of adaptability and strong stress tolerance. Most of the *Caragana* species are distributed in higher elevations and relatively harsh environments (barren, drought, heat, and cold); they are known to prevent wind and fixate sand [2–5]. Moreover, previous studies have shown that many plants have pharmacological, antibacterial, and antioxidant activities and anti-tumor, anti-HIV, and other effects [2, 5, 6]. All four species in this study have been documented in traditional Chinese ethnic medicine. Among them, *C*. *jubata* is an important Tibetan drug that can be used to treat alpine erythrocytosis and hypertension; it possesses hepatoprotective and antiviral activities[7–11].

Phylogenetic relationships in the genus *Caragana* remain obscure, and also have some problems in the identification of medicinal species. Only 10 cp-genome of the *Caragana* genus have been reported and low amount of data are available for analysis [12–14].

Chloroplasts are the posterity of ancient microbacillary endosymbionts. They are the usual organelles of green plants, which play an indispensable role in photosynthesis [15]. Ordinarily, the descendibility of the chloroplast genome is maternal in angiosperms [16]. The chloroplast genome is relatively stable in structure, and it contains a large single-copy region, small single-copy region, and two inverse repeat (IR) regions. Inverted repeat-lacking clade (IRLC) has been reported in legumes [17–20]: four *Caragana* species have been reported with IRLC [11–13]. Therefore, the *Caragana* genus can represent a lineage with extensive IRLC. However, knowledge of the pattern, origin, and evolution of plastomic IRLC within *Caragana* is presently limited by the scarcity of plastomic sequences. In addition, the chloroplast genomic model can be used to study molecular identification, phylogeny, species conservation, and evolution [21, 22].

In the present study, the four species of the genus *Caragana* from Ganzi Tibetan Autonomous Prefecture of Sichuan Province and Qinghai Province, China, were identified on the basis of the chloroplast genome. The structural characteristics, population genetics, phylogenetic relationships, and phylogenetic trees were documented.

## 2. Materials and methods

### 2.1. DNA sequencing, assembly, and validation of the chloroplast genome

The leaves of four plants were collected from Ganzi Tibetan Autonomous Prefecture of Sichuan Province and Qinghai Province, China: *C*. *jubata* location: E 97°11′18″, N 32°37′19″, altitude: 4372 m; *C*. *erinacea* location: E 98°18′13″, N 33°3′34″, altitude: 3974 m; *C*. *opulens* location: E 101°5′42″, N 31°0′8″, altitude: 2932 m; and *C*. *bicolor* location: E 100°40′26″, N 31°24′25″, altitude: 3199 m. The whole genomic DNA of *Caragana* species was extracted by using E.Z.N.A® Plant DNA kit [23]. The library was started by reagent (TruSeq™ Nano DNA Sample Prep Kit, Illumina) at 1 μg of DNA, and the DNA was interrupted to 300–500 bp by Covaris M220 ultrasound. Libraries were enriched, and eight cycles were amplified by polymerase chain reaction (PCR). Quantification was performed using TBS380 (Picogreen). Bridge PCR amplification was performed on cBot Truseq PE Cluster Kit v3-cBot-HS to generate clusters. Sequencing was conducted with 150 bp pair-end reads on the Illumina NovaSeq platform (Illumina, San Diego, CA, USA).

The cp reads were used to assemble sequences by spades, abyss, and soapdenovo. All of the contigs were aligned to the reference cp genome of *C*. *korshinskii* with MUMmer. Finally, the assembly results were inhole repaired with GapCloser-1.12 (OMEGA) [24].

### 2.2. Gene annotation and sequence analyses

Sequences were annotated by Plann [25] using the chloroplast genome of *C*. *korshinskii* from NCBI and some manual corrections. BLAST and Apollo [26] were used to check the start and stop codons and intron/exon boundaries with the cp genome of *C*. *korshinskii* as the reference sequence. The complete chloroplast genome sequence data reported in this paper have been deposited in the Genome Warehouse in National Genomics Data Center (NGDC https://ngdc.cncb.ac.cn/, accession numbers: GWHBJYO00000000, GWHBJYN00000000, GWHBJYM00000000, and GWHBJYL00000000). The structural features of the chloroplast genome were drawn by Organellar Genome DRAW [27] (http://ogdraw.mpimp-golm.mpg.de/). Protein-coding gene sequences were extracted by Geneious.

### 2.3. Comparison of chloroplast genomes

The chloroplast genomes of *Caragana* species were completed by mVISTA [28] (Shuffle-LAGAN mode) using the genome of *C*. *korshinskii* as the reference. The detecting and testing of forward, palindromic, and tandem repeats were performed using Tandem Repeats Finder [26] and REPuter [27]. In addition, the detection of simple sequence repeats (SSRs) was executed using Misa.pl [29]. The search parameters of mononucleotides, dinucleotides, trinucleotides, tetranucleotides, pentanucleotides, and hexanucleotide were set to ≥10, ≥8, ≥4, and ≥3 repeat units.

### 2.4. Phylogenetic analyses

Phylogenetic trees were constructed using plastid genomes of 18 species, in which *Ranitomeya imitator* was an outgroup. The sequences were aligned by Mafft. An unrooted phylogenetic tree with 1000 bootstrap replicates was inferred using the neighbor-joining (NJ) approach with MEGA X [30].

## 3. Results

### 3.1. DNA features of the chloroplast genome of four *Caragana* species

The size of chloroplast genome was between 127,862 and 132,780 bp, which is small because of the loss of the IR region. *C*. *opulens* (132780 bp) had the largest chloroplast genome, whereas *C*. *jubata* had the smallest (127862 bp). The mean value of the total guanine + cytosine (G+C) content of four *Caragana* species was 34.72%. Four *Caragana* species had a chloroplast genome with a similar structure, and all of them loss the IR region. After annotation, the whole chloroplast genome sequence of the four *Caragana species* was submitted to NGDC: the accession numbers are listed in Table 1.

**Table 1:** Summary of complete chloroplast genomes for four *Caragana* species.

Scample name	Total length	GC Content (%)	Depth	accession number
***C*. *jubata***	127862	34.49	749	GWHBJYN00000000
***C*. *bicolor***	131318	34.54	979	GWHBJYL00000000
***C*. *erinacea***	130968	35.15	451	GWHBJYM00000000
***C*. *opulens***	132780	34.70	992	GWHBJYO00000000

**Fig 1:**
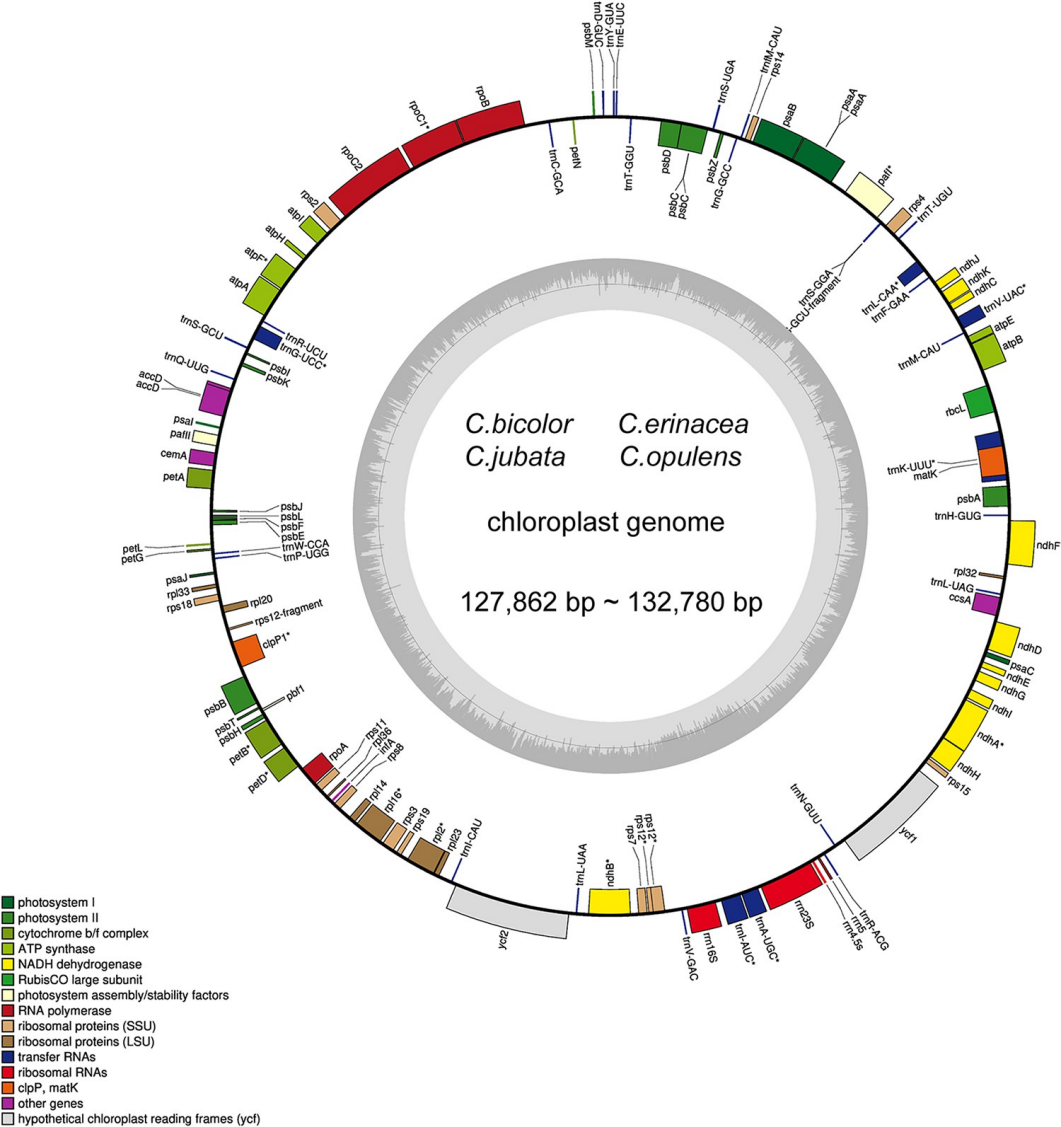
Gene map of the chloroplast genome of four *Caragana* species. Genes within the circle are transcribed clockwise, and those outside are transcribed counterclockwise. Genes belonging to different functional groups are color coded. The dark gray in the inner circle corresponds to the DNA G+C content, whereas the light-gray corresponds to the A+T content.

The gene maps of *C*. *jubata*, *C*. *bicolor*, *C*. *erinacea*, and *C*. *opulens* were drawn by OGDraw [24] (Fig 1) based on annotation results. A total of 112 genes were found in the chloroplast genome of *C*. *jubata*, *C*. *bicolor*, *C*. *erinacea*, and *C*. *opulens*: 30 tRNA, 4rRNA, and 78 protein-coding genes. Among the 112 genes, 43 were photosynthesis-related genes (Table 2). Most genes could be divided crudely into three groups: “photosynthesis-related,” “self-replication-related,”, and “other” groups (Table 2) [31].

**Table 2:** Genes in the chloroplast genome of four *Caragana* species.

Category	Group of genes	Name of genes
Self-replication	Large subunit of ribosomal proteins	*Rp114*, *16*, *2*, *20*, *22*, *23*, *32*, *33*, *36*
	Small subunit of ribosomal proteins	*rps2*, *3*, *4*, *7*, *8*, *11*, *12*, *14*, *15*, *18*, *19*
	DNA-dependent RNA polymerase	*rpoA*, *B*, *C1*, *C2*
	rRNA genes	*rrn16S*^*a*^, *rrn23S*^*a*^, *rrn4*.*5S*^*a*^, *rrn5S*^*a*^
	tRNA genes	*trnA-UGC*, *trnC-GCA*, *trnD-GUC*, *trnE-UUC*, *trnF-GAA*, *trnfM-CAU*, *trnG-GCC*, *trnG-UCC*, *trnH-GUG*, *trnI-AUC*, *trnI-CAU*, *trnK-UUU*, *trnL-CAA*, *trnL-UAA*, *trnL-UAG*, *trnM-CAU*, *trnN-GUU*, *trnP-UGG*, *trnQ-UUG*, *trnR-ACG*, *trnR-UCU*, *trnS-GCU*, *trnS-GGA*, *trnS-UGA*, *trnT-GGU*, *trnT-UGU*, *trnV-GAC*, *trnV-UAC*, *trnW-CCA*, *trnY-GUA*
Photosynthesis	Photosystem I	*psaA*, *B*, *C*, *I*, *J*
	Photosystem II	*psbA*, *B*, *C*, *D*, *E*, *F*, H, *I*, *J*, *K*, *L*, *M*, *T*, *Z*,
	NADH oxidoreductase	ndhA^*^, B^*,*a*^, *C*, *D*, *E*, *F*, *G*, *H*, *I*, *J*, *K*
	Cytochrome b6/f complex	*petA*, B^*^, D^*^, *G*, *L*, *N*
	ATP synthase	*atpA*, *B*, *E*, F^*^, *H*, *I*
	Rubisco	*rbcL*
Other genes	Maturase	*matK*
	Protease	*clpP* ^*^
	Envelope membrane protein	*cemA*
	Subunit acetyl-CoA-carboxylase	*accD*
	c-Type cytochrome synthesis gene	*ccsA*
	Conserved open-reading frames	*Ycf1*, *2*
	protein synthesis initiation factor	*infA*

Of the anticipated genes of the chloroplast genomes of *C*. *jubata*, *C*. *bicolor*, *C*. *erinacea*, and *C*. *opulens*, introns were discovered in 17 genes: six tRNA(*trnV-UAC*, *trnL-CAA*, *trnG-UCC*, *trnA-UGC*, *trnK-UUU*, and *trnI-AUC*) genes and 11 protein-encoding genes (*rpl2*, *rps12*, *rpoC1*, *rpl16*, *ndhA*, *ndhB*, aptF, *clpP*, *petB*, *pafI*, and *petD*; Table 3). Most of the 17 intron-containing genes were inserted by one intron except for *pafI*, which was inserted by two introns. In the four cp-genomes, the longest intron all were *trnH-UUU*, which in length (2481, 2494, 2494, and 2485 bp), and contained the whole *matK*.

**Table 3:** The genes having introns in the chloroplast genome of four *Caragana* species and the length of the exons and introns.

Species	Gene	Exon I (bp)	Intron I (bp)	Exon II (bp)	Intron II (bp)	Exon III (bp)
*C*. *jubata*	*trnL-CAA*	37	350	50		
	*trnV-UAC*	39	574	37		
	*trnA-UGC*	38	812	35		
	*trnG-UCC*	21	682	51		
	*trnI-AUC*	37	953	35		
	*trnK-UUU*	37	2481	29		
	rps12*	26	232	591		
	*rpl16*	399	1105	9		
	*rpl2*	434	689	394		
	*rpoC1*	430	790	1625		
	*ndhA*	553	1163	539		
	*ndhB*	762	685	777		
	*aptF*	145	702	410		
	*petB*	6	819	642		
	*clpP*	231	786	292		
	*petD*	8	717	475		
	*pafI*	124	711	230	886	153
*C*. *erinacea*	*trnL-CAA*	37	555	50		
	*trnV-UAC*	39	572	37		
	*trnA-UGC*	38	808	35		
	*trnG-UCC*	21	679	51		
	*trnI-AUC*	37	956	35		
	*trnK-UUU*	37	2494	29		
	rps12*	26	232	588		
	*rpl16*	399	945	9		
	*rpl2*	434	689	388		
	*rpoC1*	430	785	1625		
	*ndhA*	551	1194	541		
	*ndhB*	723	680	762		
	*petB*	6	822	642		
	*clpP*	363	843	231		
	*petD*	8	729	475		
	*aptF*	145	697	410		
	*pafI*	124	714	230	891	153
*C*. *bicolor*	*trnV-UAC*	39	572	37		
	*trnL-CAA*	37	555	50		
	*trnK-UUU*	37	2494	29		
	*trnI-AUC*	37	956	35		
	*trnA-UGC*	38	808	35		
	*rps12*	26	232	588		
	*rpoC1*	430	785	1625		
	*rpl2*	390	686	435		
	*rpl16*	399	945	9		
	*petD*	8	729	475		
	*petB*	6	822	642		
	*pafI*	126	714	226	981	155
	*ndhB*	723	680	762		
	*ndhA*	551	1194	541		
	*clpP1*	363	843	231		
	*atpF*	167	673	412		
*C*. *opulens*	*trnV-UAC*	39	574	37		
	*trnL-CAA*	37	534	50		
	*trnK-UUU*	37	2485	29		
	*trnI-AUC*	37	953	35		
	*trnG-UCC*	21	682	51		
	*trnA-UGC*	38	810	35		
	*rps12*	26	232	600		
	*rpoC1*	430	789	1625		
	*rpl2*	396	692	435		
	*rpl16*	399	1101	9		
	*petD*	8	720	475		
	*petB*	6	826	642		
	*pafI*	126	702	226	873	155
	*ndhB*	723	685	762		
	*ndhA*	551	1169	541		
	*clpP1*	363	1628	223		
	*atpF*	167	679	412		

### 3.2 Analyses of long repetitive sequences and SSRs

**Fig 2:**
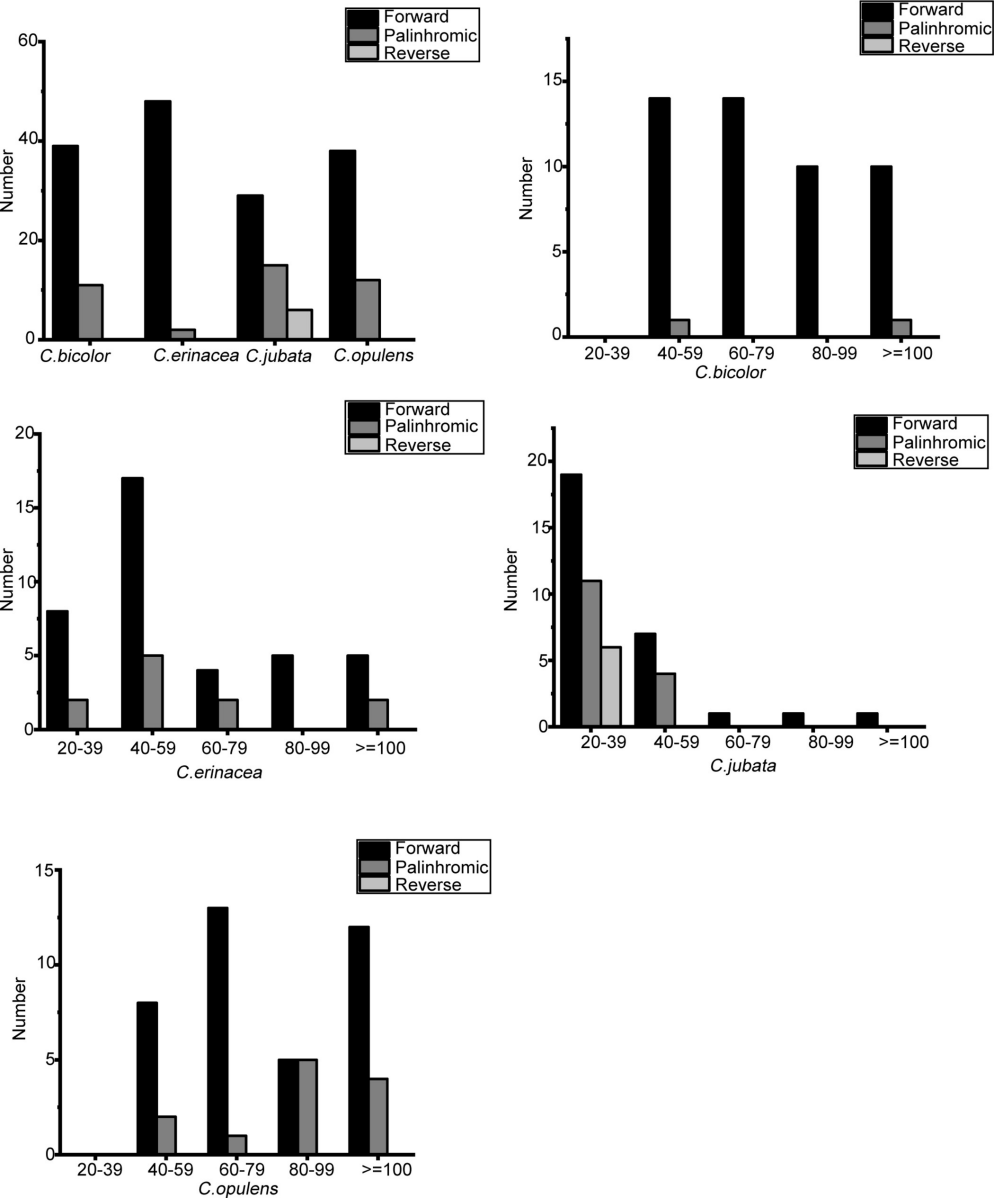
Long repetitive sequences in chloroplast genomes of four *Caragana* species.

For *C*. *jubata*, *C*. *bicolor*, *C*. *erinacea*, *and C*. *opulens*, interspersed repeated sequences (IRSs) were evaluated in the chloroplast genome with a repeat-unit length of ≥ 20 bp. These sequences comprised only forward reverse and palindromic repeats, yet they lacked complementary repeats that are common in other species. Among them, a total of 50 IRSs were found. Among all types of IRS, the sequence lengths in the range of 20–39 bp occurred most frequently in *C*. *jubata* and 40–59 bp occurred most frequently in *C*. *erinacea*. Those in the range of 60–79 bp and ≥ 100 bp occurred most frequently in *C*. *opulens*. IRS analyses of chloroplast genomes are shown in Fig 2.

**Fig 3:**
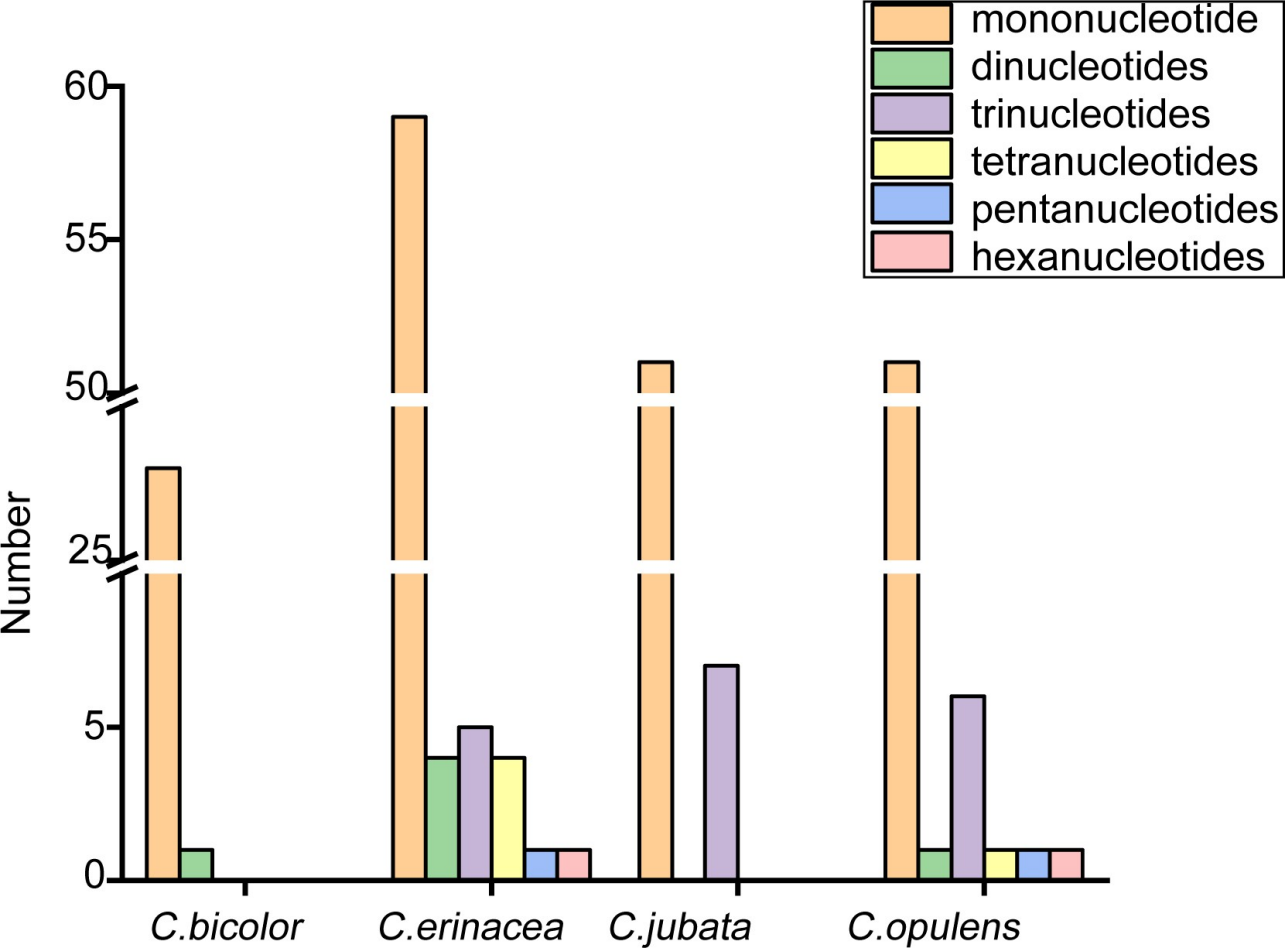
SSR distribution in chloroplast genomes of four Caragana species.

The key mutational mechanism generating SSR polymorphism is as follows: SSRS tended to undergo slipped-strand mispairing [32]. However, SSRs in chloroplast genomes are often used as genetic markers in evolutionary and population genetic studies because of their variability at the intra-specific level [33, 34]. We found 58 SSRs in *C*. *jubata*, 29 SSRs in *C*. *bicolor*, 61 SSRs in *C*. *opulens*, and 74 SSRs in *C*. *erinacea* (Fig 3).

### 3.3. Comparative genomic analysis

**Fig 4:**
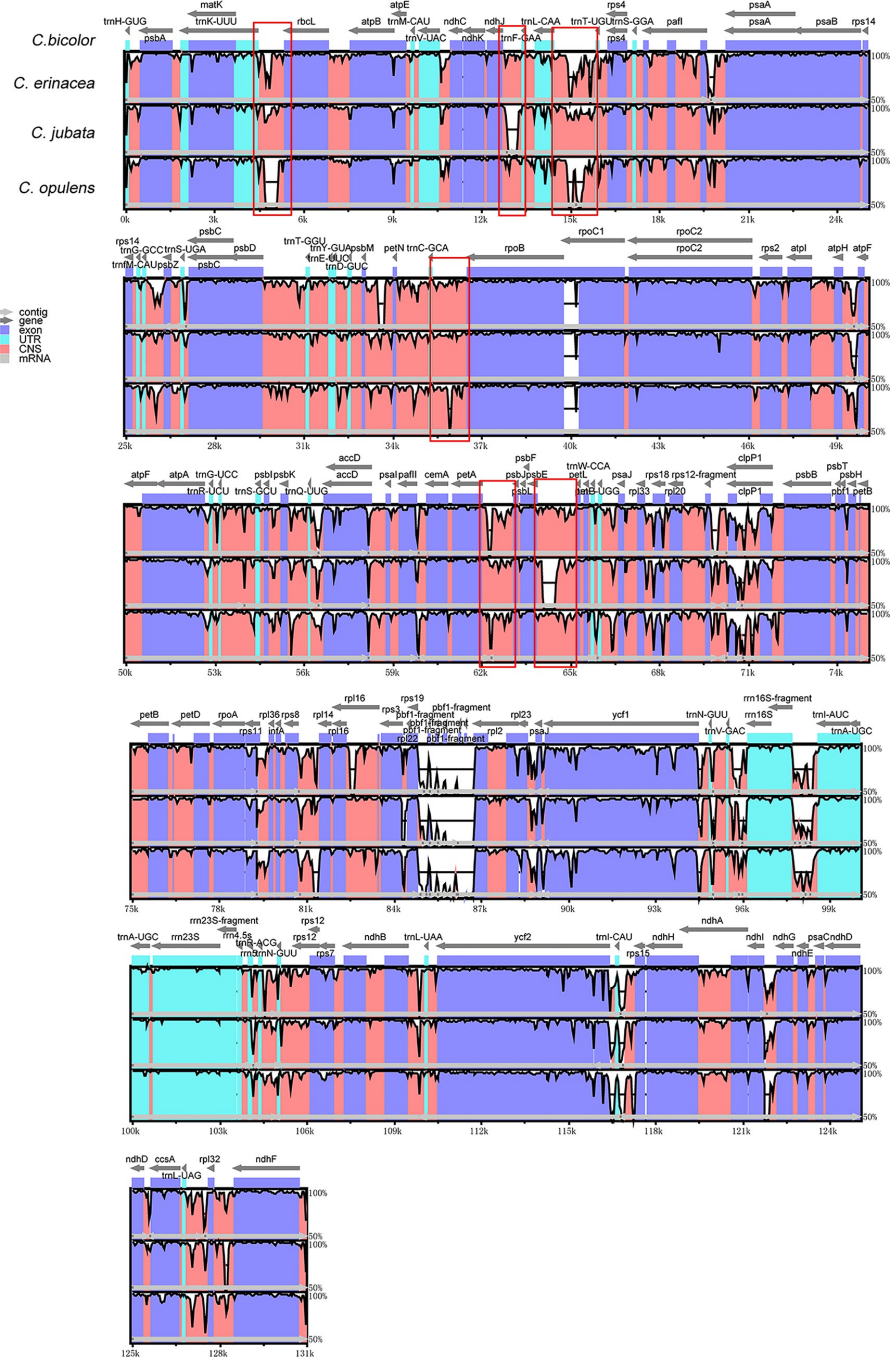
Comparative analyses of genomic differences in chloroplasts of four *Caragana* species. Gray arrows and thick black lines above the alignment indicate gene orientation. Purple bars represent exons; blue bars denote untranslated regions (UTRs); pink bars represent non-coding sequences (CNS), and gray bars denote mRNA. The y-axis represents percentage identity.

In elucidating the differences in genomic sequences of *C*. *jubata*, *C*. *erinacea*, *C*. *opulens*, and *C*. *bicolor*, we used mVISTA to detect sequence variations using the sequence in *C*. *bicolor* as a reference (Fig 4). The four genomic sequences are highly similar. However, in some intergenic spacer (IGS) regions and partial sequences, significant differences are found, such as the IGS between *trnK-UUU* and *rbcl*; *trnF-GAA* and *ndhJ*; *trnL-CAA* and *trnT-UGU*; *rpoB* and *trnC-GCA*; *petA* and *psbL*; *psbE* and *pebL*; and sequences of the *rpoC*, *ycf1*, and *ycf2*. The noncoding regions have different degrees of divergence, whereas the protein coding regions are highly conserved. This finding indicated that the IGS of the *Caragana* genus evolved rapidly. Highly variable regions can be used to exploit markers for identification and further phylogenetic study.

### 3.4 Phylogenetic analyses

**Fig 5:**
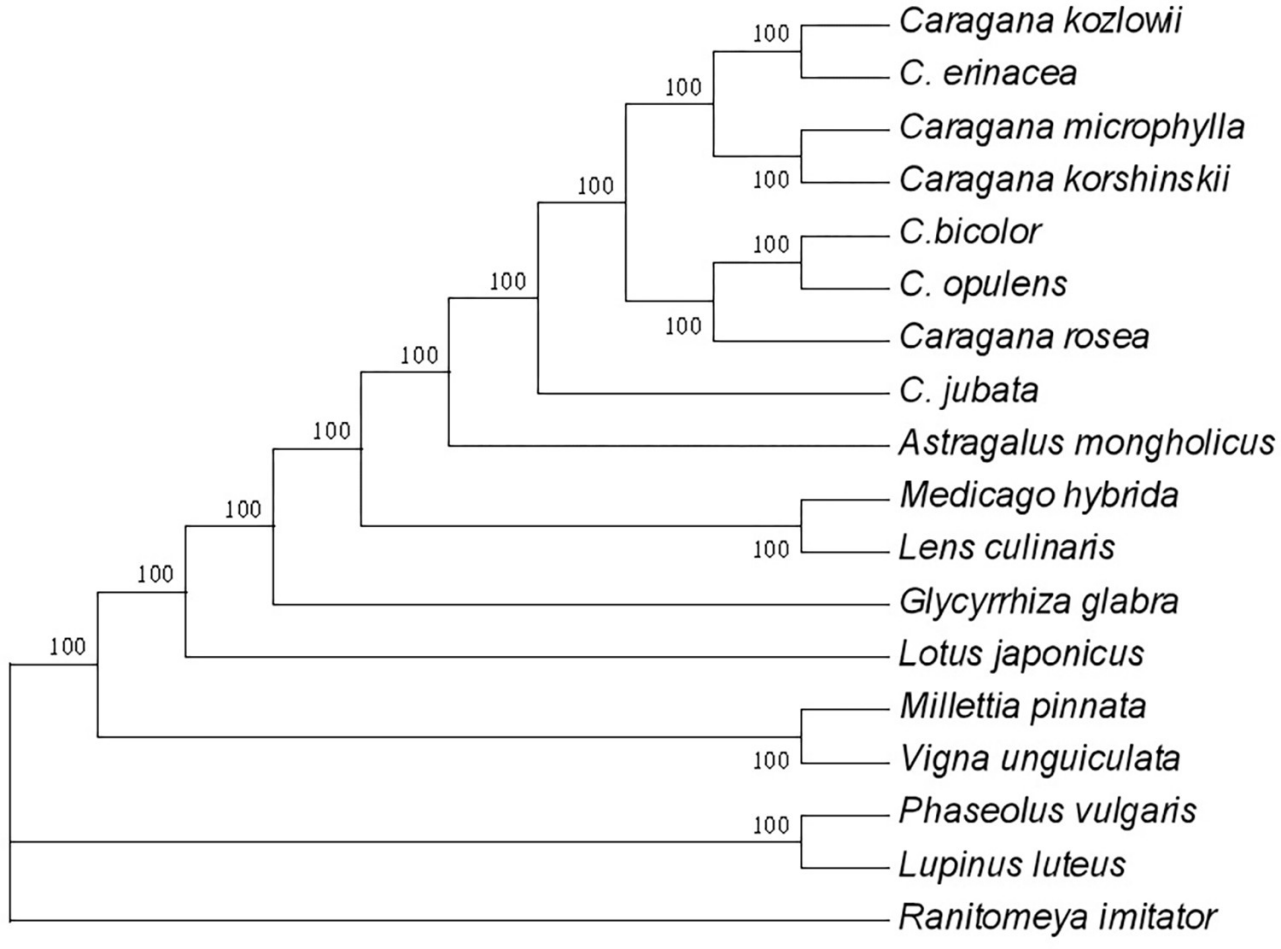
NJ tree based on chloroplast genomes of the Fabaceae family.

In determining the phylogenetic position of *Caragana* species, 18 complete chloroplast genome sequences of the Fabaceae family were constructed using the NJ tree (Fig 5). The other 14 species belong to *Lens*, *Medicago*, *Caragana*, *Astragalus*, *Glycyrrhiza*, *Lotus*, *Millettia*, *Vigna*, *Phaseolus*, *Lupinus*, and *Mimosa*.

The results showed that eight species from *Caragana* were relatives and categorized together. The following pairs showed a closer relationship: *C*. *kozlowii* and *C*. *erinacea*, *C*. *microphylla* and *C*. *korshinskii*, and *C*. *opulens* and *C*. *bicolor*. The genera *Astragalus* and *Caragana* were classified into the *Subtrib*. Astragalinae: *C*. *jubata* belongs to *Ser*. *Jubatae*; *C*. *bicolor* belongs to *Ser*. *Occidentales*; *C*. *erinacea* belongs to *Ser*. *Spinosae*, and *C*. *opulens* belongs to *Ser*. *Grandiflorae Pojark*^.^ [1]. This result also fitted the clustering results based on ITS2 sequences [35].

## 4. Conclusions and discussion

The chloroplast genomes of 10 species of the genus *Caragana* have been published in the National Center for Biotechnology Information; chloroplast genomes were between 127,103 and 133,122 bp in size, and they contained 110–111 genes (30–31 tRNA, 4 rRNA, and 76 protein-coding genes). Multiple species of *Caragana* have been reported to loss the IR region, such as *C*. *rosea*, *C*. *microphylla*, and *C*. *intermedia*[12–14]. The chloroplast genomes of *C*. *jubata*, *C*. *erinacea*, *C*. *opulens*, and *C*. *bicolor* showed high similarity with regard to gene deletion, genome size, gene sequences, gene classes, and distribution of repeat sequences, and the lacked IRLC. An important indicator of species affinity is the content of DNA G + C [36], and the four *Caragana* species in this study have highly similar cpDNA G+C content.

IRS analyses of chloroplast genomes show that the four species lacked complementary repeats. Comparative cp-genomic analysis suggested that the cp genome structure of *Caragana* was well conserved. Highly variable regions are primarily distributed in non-coding and partial coding regions, which can be used to exploit markers for identification and further phylogenetic study.

Intron and/or gene losses in chloroplast genomes have been reported in considerable literature [37–39]. Introns can play an important role in the regulation of gene expression in a temporal and tissue-specific manner [39–41]. Regulatory mechanisms of introns in some plants and animals have been reported [42–44]. However, the relationship between intron deletion and gene expression in *Caragana* by transcriptome has not been published. Therefore, further research into the role of introns in *Caragana* species is necessary.

Advances in phylogenetic analysis can reveal the evolution of chloroplast genomes, including nucleotide substitutions and structural changes [45, 46]. Our results of phylogenetic analysis were consistent with the status of the major taxa within the genus *Caragana* [1]. Species from the genus *Caragana* were monophyletic, and *C*. *jubata*, *C*. *erinacea*, *C*. *opulens*, and *C*. *bicolor* could be differentiated from other *Caragana* species. The current study demonstrated that chloroplast genomes can be used for the identification and classification of *Caragana* species. In addition, the phylogenetic relationship of *Caragana* species is related to elevation and geographical distribution (GD). For example, *C*. *kozlowii* (altitude: 3100–4300 m; GD: Qinghai, E Xizang) and *C*. *erinacea* (altitude: 2000–4000 m, GD: Qinghai, Xizang, Gansu, Ningxia, W Sichuan, NW Yunnan) are distributed at altitudes of 3100–4000 levels in Qinghai and Tibet. In addition, *C*. *microphylla* (altitude: 1800–3800 m, GD: Nei Mongol, Jilin, Liaoning) and *C*. *korshinskii* (altitude: 900–2400 m, GD: Nei Mongol, Gansu, Ningxia, Qinghai, W Shanxi) are distributed at altitudes of 1800–2400 levels in Nei Mongo. The phylogenetic relationship of *Caragana* species partially consistent with elevation and GD. This finding may provide a reference for further study on the relationship between distribution and evolution of *Caragana* species.

*Caragana* species have a large altitude span and wide GD, showing strong environmental adaptability[1–5]. Our results can provide valuable information for genetic transformation, the development of population genetic surveys, and evolutionary studies. Plastids contain a range of genes associated with photosynthesis, and photosystem II is a key component of high temperature, drought stress, and many other stresses [47, 48]. However, the strong environmental adaptability mechanism of the genus *Caragana* remains unclear because of the lack of research and data[3, 12]. Our results can provide data for further investigation of the discovery of adaptability and strong adversity resistance genes of *Caragana* species. Our research data complement the database of herbgenomics [49, 50].
